# Apoptosis Inhibitor of Macrophage (AIM) Modulates Calcium Oxalate-Induced Ureteral Fibrosis in AIM-Felinized Mice

**DOI:** 10.3390/ijms26189117

**Published:** 2025-09-18

**Authors:** Yuka Machida, Masaki Watanabe, Fumi Suzuki, Ryo Ando, Koudai Watanabe, Yugo Moriya, Kenichi Maeda, Shozo Okano, Tadashi Okamura, Ryoichi Sugisawa, Nobuya Sasaki, Satomi Iwai

**Affiliations:** 1 Laboratory of Small Animal Surgery 2, School of Veterinary Medicine, Kitasato University, Aomori 034-8628, Japan; 2 Laboratory of Laboratory Animal Science, School of Veterinary Medicine, Kitasato University, Aomori 034-8628, Japan; 3 Laboratory of Veterinary Pathology, School of Veterinary Medicine, Kitasato University, Aomori 034-8628, Japan; 4 Department of Laboratory Animal Medicine, National Institute of Global Health and Medicine, Japan Institute for Health and Security, Tokyo 162-8655, Japan; 5 Department of Biochemistry, Kindai University Faculty of Medicine, Osaka 589-8511, Japan

**Keywords:** feline ureteral obstruction, apoptosis inhibitor of macrophage, mouse mode

## Abstract

Calcium oxalate (CaOx) stones account for 90% of uroliths in cats and contribute to ureteral inflammation and fibrosis, although the underlying mechanism remains unclear. Apoptosis inhibitor of macrophage (AIM) is known to play a protective role against tubular injury in feline kidney disease. This study investigated whether AIM contributes to ureteral fibrosis by using AIM-felinized mice subjected to CaOx bead-induced ureteral injury. Male C57BL/6 mice (*n* = 54), including wild-type mice (mA), AIM-knockout (koA) mice, and AIM-felinized mice (fA), were assigned to either a unilateral ureteral obstruction (UUO; U) group or a UUO plus CaOx implantation (C) group. Ureters were collected 14 days after the procedure for histopathological analysis. The severity of ureteral injury followed the order of koA-C ≥ fA-C > mA-C, indicating AIM’s involvement in the injury process. Furthermore, fA exhibited more severe fibrosis than mA mice (*p* < 0.05), suggesting that mouse AIM may have stronger anti-fibrotic effects than feline AIM. These results suggest that AIM-felinized mice could serve as a useful model for investigating feline-specific ureteral pathology. To our knowledge, this is the first experimental study to explore the role of feline AIM in ureteral injury and fibrosis. Further studies are warranted to validate the utility of this model.

## 1. Introduction

Ureteral obstruction in cats is increasingly recognized in veterinary medicine [1,2,3,4,5]. Causes of obstruction include uroliths, blood clots, inflammatory products, tissue fragments, tumors, trauma, fibrosis, and emboli composed of minerals and matrix substances [6,7,8]. Ureterolithiasis accounts for approximately 72–87% of ureteral obstructions [5,9,10], with the most common types of stones being calcium oxalate (CaOx) and magnesium ammonium phosphate (struvite). Since 2000, the incidence of CaOx stones has risen [1], now accounting for more than 90% of upper urinary tract uroliths [2]. CaOx stone formation is often associated with hypercalciuria due to age-related changes in the kidneys and is thus commonly observed in older cats. However, the widespread use of therapeutic diets for struvite stones has led to an increase in CaOx stones among younger cats as well [5]. Unlike struvite stones, CaOx stones are insoluble and cannot be managed through diet induced dissolution [11].

Currently, treatment options include surgical reconstruction, such as ureterostomy, ureterectomy with anastomosis, ureteral stenting, and subcutaneous ureteral bypass (SUB) system placement [12]. However, owing to the anatomical features of the feline ureter and the technical difficulty of surgical reconstruction, facilities capable of performing such procedures are limited. The SUB system is easier to perform than other surgical methods and is widely used, but complications such as chronic urinary tract infections, tube migration, and system re-obstruction have been reported [13,14]. These issues have led to increased postoperative management burdens and a decline in the popularity of the procedure. As stated in the ACVIM guidelines [15], medical management is not currently recommended due to the risk of decreasing renal function, and no effective medical treatment is available. Ureterolithiasis causes both functional and morphological changes in the ureter. Typical changes include proximal dilation due to obstruction, chronic granuloma formation from stone migration, fibrosis, and the potential development of ureteral strictures. These morphological changes are accompanied by reduced peristalsis in the ureteral smooth muscle [7,16].

The extent of ureteral damage caused by ureterolithiasis in cats is believed to differ from that in dogs, humans, or mice. In cats, the ureteral diameter is approximately 0.4 mm [17], narrower than in dogs. During feline ureterolithiasis surgery, peristalsis is rarely observed, whereas it is often retained in dogs, allowing small stones to be flushed into the bladder. Once a stone forms in a cat, it tends to remain in the ureter due to its narrow diameter.

Furthermore, feline tissue healing is more prone to granulation tissue formation and is considered less regenerative than in dogs [18]. As the mechanism of ureteral injury in cats differs from that of other species and remains largely unclear, no treatments are currently available to prevent fibrosis or restore ureteral function.

In previous studies, ureteral obstruction models have been created in mice or rats by surgical ligation of the ureter [19,20]. In addition, our group developed a mouse model by implanting CaOx beads into the ureteral lumen following ureteral ligation to directly induce injury and evaluate ureteral lumen damage caused by calcium oxalate stones [21]. The results showed ureteral inflammation, such as infiltration of degenerative neutrophils, appearance of foreign body giant cells, and hyperplasia of transitional epithelium. Additionally, features of fibrosis such as granulation tissue formation, epithelial desquamation, and connective tissue extension to the muscular layer were observed [21]. However, due to species differences in ureteral injury between mice and cats, these mouse models cannot be directly applied to feline ureteral obstruction. Therefore, an appropriate feline ureteral obstruction model is needed to elucidate the mechanism of obstruction, investigate stone treatments, and develop therapies for ureteral injury.

In 1999, Toru Miyazaki and colleagues discovered Apoptosis Inhibitor of Macrophage (AIM, also called CD5L) [22]. AIM is a circulating blood protein produced by macrophages that forms an inactive complex with IgM pentamer in the bloodstream [23,24,25,26,27]. When AIM becomes active by dissociating from IgM, it binds to various biological wastes such as cellular debris and Damage-Associated Molecular Patterns (DAMPs), marking them for enhanced phagocytosis by macrophages [28,29,30,31,32,33,34]. This mechanism of AIM plays various roles, including suppressing obesity, fatty liver, and liver cancer progression [27,31,35,36,37,38,39,40,41]. AIM has also been shown to be involved in diseases such as kidney disease, cerebral infarction, and peritonitis, and its supplementation has been demonstrated to prevent and treat these conditions [32,42,43,44,45,46,47,48,49,50].

It has been reported that feline AIM differs from that of humans and mice [41,51]. In acute kidney injury (AKI), AIM promotes self-repair in humans and mice [33], but in cats, this recovery mechanism is impaired [51]. Normally, AIM bound to IgM cannot pass through the glomerular filtration barrier. However, during AKI, AIM in humans and mice dissociates from IgM, passes through the glomerulus, and binds to debris in the proximal tubules, facilitating phagocytosis by tubular epithelial cells and promoting early recovery [33]. In contrast, feline AIM has a 1000-fold higher affinity for IgM than that of mice [51], preventing its dissociation during AKI and thereby blocking filtration into primary urine. As a result, recovery is hindered, and fibrosis progresses, leading to chronic disease.

Moreover, AIM-felinized mice show higher mortality from AKI compared to wild-type mice, whereas administration of recombinant AIM improves both renal function and survival [51]. If recovery from AKI is impaired, it can easily progress to chronic kidney disease (CKD), suggesting that feline AIM may significantly influence mortality from kidney disease. Based on these findings, we hypothesized that AIM may be involved in the species differences observed in ureteral injury and fibrosis caused by feline ureterolithiasis. Therefore, the objective of this study was to create a ureteral obstruction model using CaOx bead implantation in AIM-felinized mice and to investigate whether this mouse model can serve as a suitable analog for feline ureteral obstruction caused by urolithiasis. While the role of AIM in feline kidney disease has been previously reported, no studies have investigated its involvement in feline ureteral injury. Given the species-specific behavior of feline AIM, we hypothesized that it may play a pivotal role in ureteral fibrosis following obstruction. To our knowledge, this is the first study to explore the effect of feline AIM on ureteral damage using a genetically engineered mouse model.

## 2. Results

### 2.1. Blood Biochemistry Test

**Figure 1:**
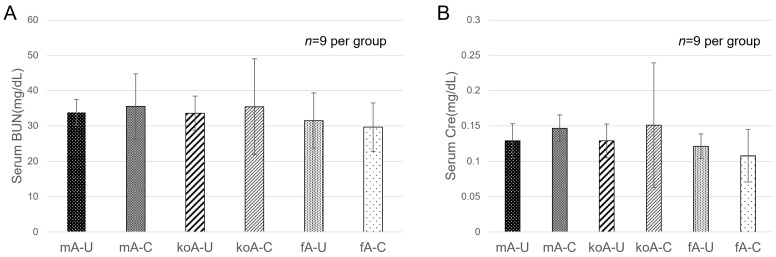
(**A**) Serum BUN levels and (**B**) serum creatinine (Cre) levels measured at 2 weeks after CaOx bead implantation (*n* = 9 per group). Data were compared using one-way ANOVA (BUN) and Kruskal–Wallis (Cre) test. No significant differences were observed among the groups.

In terms of blood urea nitrogen (BUN), a marker of kidney function, the values were as follows: fA-C group (29.6 ± 6.84 mg/dL), fA-U group (31.5 ± 7.83 mg/dL), koA-C group (35.4 ± 13.57 mg/dL), koA-U group (33.6 ± 4.88 mg/dL), mA-C group (35.5 ± 9.24 mg/dL), and mA-U group (33.8 ± 3.65 mg/dL). No significant differences were observed among the groups (Figure 1A). For creatinine (Cre), the values were as follows: fA-C (0.11 ± 0.04 mg/dL), fA-U (0.12 ± 0.02 mg/dL), koA-C (0.15 ± 0.09 mg/dL), koA-U (0.13 ± 0.02 mg/dL), mA-C group (0.14 ± 0.02 mg/dL), and mA-U group (0.13 ± 0.02 mg/dL). Again, no significant differences were observed among the groups (Figure 1B).

### 2.2. Histopathological Findings

**Figure 2:**
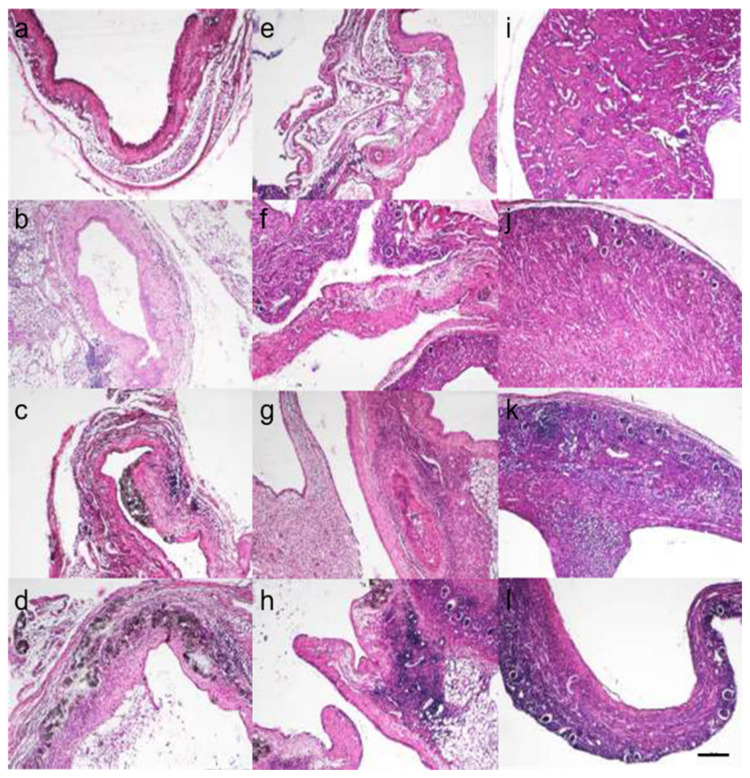
Evaluation of inflammatory cell infiltration in HE-stained sections. The scale bar represents 200 µm. All images were captured at the same magnification. (Representative images used for quantification in each group are shown in Supplementary Figure S1). Representative images of inflammatory cell infiltration in the ureter (**a**–**d**), renal pelvis (**e**–**h**), and renal parenchyma (**i**–**l**) were classified into four grades: Grade 0, no infiltration (**a**,**e**,**i**); Grade 1, slight infiltration (**b**,**f**,**j**); Grade 2, moderate or focal infiltration (**c**,**g**,**k**); Grade 3, extensive infiltration (**d**,**h**,**l**).

**Figure 3:**
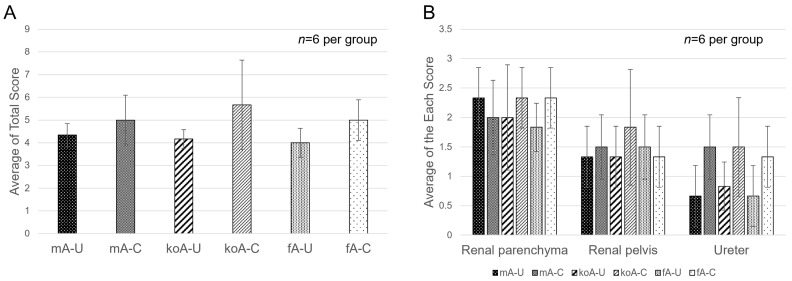
(**A**) Total scores and (**B**) individual scores for inflammatory cell infiltration in the renal parenchyma, renal pelvis, and ureter, assessed based on hematoxylin and eosin (HE) staining (*n* = 6 per group). Data were compared using one-way ANOVA. No significant differences were observed among the groups.

**Figure 4:**
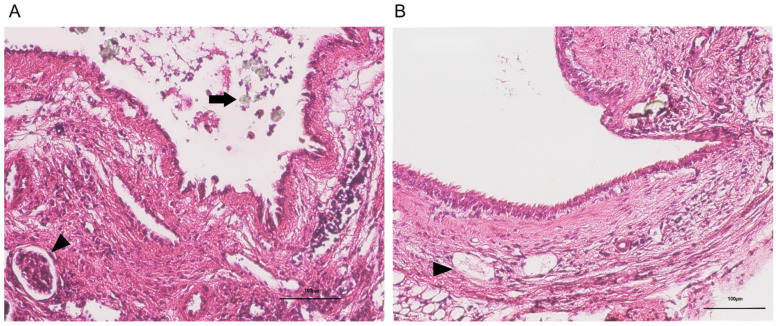
Representative HE-stained images (×100) of ureteral tissue from the fA–C (**A**) and mA–U (**B**) groups (*n* = 6 each). The scale bar represents 100 µm. (**A**) Slight inflammatory cell infiltration and vascular dilation (arrowheads), and exfoliation of the urothelium caused by CaOx beads (arrows) are observed. (**B**) No urothelial exfoliation is detected. (Representative images used for quantification in each group are shown in Supplementary Figure S2).

Using Hematoxylin and Eosin (HE) stained tissue sections, we evaluated the infiltration of inflammatory cells, the shedding of the transitional epithelium, and vascular dilation. The degree of inflammatory cell infiltration in the renal parenchyma, renal pelvis, and ureter was graded from 0 to 3 (Figure 2: no cases were classified as Grade 4), and the total score for the three regions was averaged. The results are shown in Figure 3A,B. The results were as follows: fA-C group (5.00 ± 0.89), fA-U group (4.00 ± 0.63), koA-C group (5.67 ± 1.97), koA-U group (4.17 ± 0.41), mA-C group (5.00 ± 1.10), and mA-U group (4.33 ± 0.52), and no significant differences were observed among the groups. In the renal parenchyma, Grade 3 infiltration was frequently observed in the CaOx group, with diffuse infiltration of neutrophils and lymphocytes in the interstitium. In the ureter, Grade 1 to 2 infiltration was observed regardless of the group. Additionally, while some cases in the CaOx groups showed transitional epithelial cell loss in the ureter, many cases exhibited accumulation of CaOx beads in the dilated renal pelvis. Vasodilation was observed in the blood vessels of the ureter and renal pelvis across all groups (Figure 4A,B).

### 2.3. Quantification of Fibrosis

**Figure 5:**
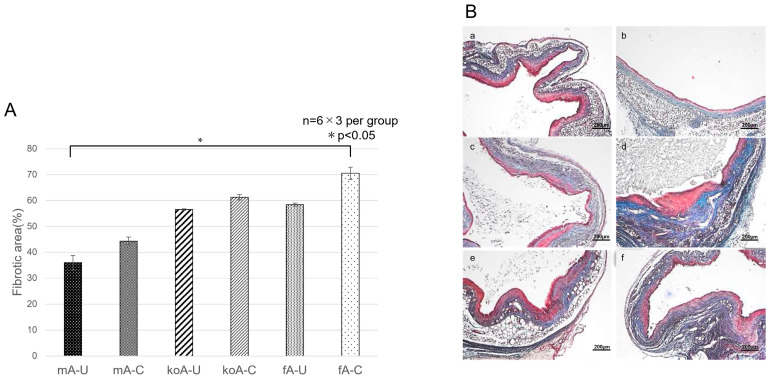
(**A**) Comparison of fibrotic area (%) among experimental groups. Data were compared using Kruskal–Wallis test followed by pairwise comparisons with the Steel–Dwass test (*n* = 6 per group, 3 fields per sample). (**B**) Representative Masson’s trichrome (MT)-stained microscopic images (×40) of ureteral tissue from each group (*n* = 6 per group). Panels: (**a**), mA-U group; (**b**), mA-C group; (**c**), koA-U group; (**d**), koA-C group; (**e**), fA-U group; (**f**), fA-C group. The scale bar represents 200 µm.

Using Masson’s Trichrome (MT) stained tissue sections, we quantified fibrosis using Image J. The percentages of fibrotic areas were as follows: fA-C (70.56 ± 2.38%), fA-U (58.42 ± 0.58%), koA-C (61.25 ± 1.00%), koA-U (56.47 ± 0.29%), mA-C (44.36 ± 1.50%), and mA-U (35.94 ± 2.75%). Statistically significant differences (*p* < 0.05) were found between the fA-C vs. mA-U (*p* = 0.0333) (Figure 5A,B).

### 2.4. Immunohistochemistry (IHC) Staining

#### 2.4.1. αSMA (Acta2)

**Figure 6:**
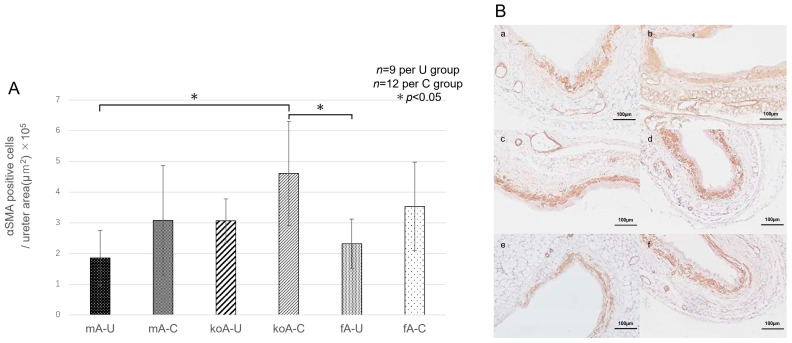
(**A**) Proportion of αSMA-positive cells relative to the ureteral area (*n* = 18). Data were compared using Kruskal–Wallis test followed by pairwise comparisons with the Steel–Dwass test (*n* = 6 per group, 3 fields per sample). Significant differences were observed (* *p* < 0.05). (**B**) Representative immunohistochemically stained microscopic images (×40) of ureteral tissue using a rabbit polyclonal anti-αSMA antibody (*n* = 3 per group). Panels: (**a**), mA-U group; (**b**), mA-C group; (**c**), koA-U group; (**d**), koA-C group; (**e**), fA-U group; (**f**), fA-C group. The scale bar represents 100 µm. Quantification is shown as the number of αSMA positive cells per ureter area (µm^2^), multiplied by 10^5^ for clarity.

Sections stained with a rabbit polyclonal anti-αSMA antibody were imaged, and the proportion of αSMA-positive cells relative to the ureteral area was calculated. The results were as follows: fA-C group (3.53 × 10^−5^ ± 1.44), fA-U group (2.32 × 10^−5^ ± 0.80), koA-C group (4.60 × 10^−5^ ± 1.70), koA-U group (3.07 × 10^−5^ ± 0.71), mA-C group (3.08 × 10^−5^ ± 1.78), and mA-U group (1.86 × 10^−5^ ± 0.89). Statistically significant differences (*p* < 0.05) were observed between the following groups: koA-C vs. mA-U (*p* = 0.0195), and koA-C vs. fA-U (*p* = 0.0375) (Figure 6A,B).

#### 2.4.2. AIM (CD5L)

**Figure 7:**
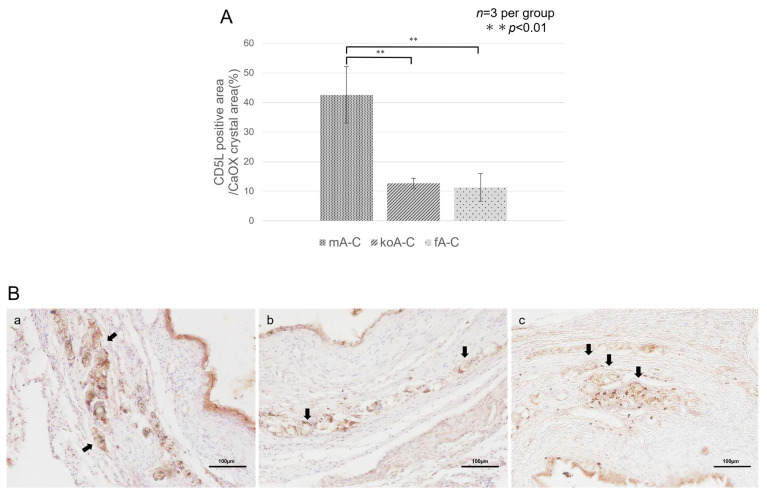
(**A**) Proportion of AIM (CD5L)-positive areas in the CaOx stone area as determined by immunohistochemical staining using a rabbit polyclonal anti-AIM antibody (*n* = 3 per group, 3 fields per sample). Data were compared using one-way ANOVA, followed by the Tukey–Kramer post hoc test (** *p* < 0.01). (**B**) Representative microscopic images (×100) of ureteral tissue stained by IHC using the same antibody (*n* = 3 per group). Panels: (**a**), mA-C group; (**b**), koA-C group; (**c**), fA-C group. AIM-positive areas are observed to accumulate around CaOx stones within the ureteral tissue (arrows: CaOx stones). The scale bar indicates 100 µm.

Tissue sections stained with rabbit polyclonal anti-CD5L antibody were used to assess the AIM-positive area relative to the CaOx crystal area using ImageJ. The percentages were: fA-C (11.3 ± 4.70%), koA-C (12.71 ± 1.69%), and mA-C (42.60 ± 9.54%). Statistically significant differences (*p* < 0.01) were observed between the mA-C and fA-C groups (*p* = 0.0057) and between the mA-C and koA-C groups (*p* = 0.0071). Microscopic images showed that AIM tended to accumulate around CaOx crystals, which were prominent in the mA-C group, whereas AIM-positive areas were scarcely observed around the crystals in the fA-C and koA-C groups (Figure 7A,B).

## 3. Discussion

Most disease models of ureteral obstruction have focused on its impact on the kidneys, while few studies have examined the ureter specifically. Furthermore, existing ureter-focused models have involved external damage such as ligation, and only one previous study has established a model of intraluminal injury [21]. However, the pathological characteristics of feline ureteral obstruction differ from those in mice, and a feline-specific model of ureteral obstruction has yet to be developed. Therefore, the present study focused on AIM, a protein known to behave differently in mice and cats under pathological conditions, and aimed to create a new model of intraluminal ureteral injury using AIM-felinized mice to investigate the role of AIM in this pathology. AIM is a blood protein produced by tissue macrophages, and under normal conditions, its circulates are bound to pentameric IgM [23,24,25,26,27]. This binding prevents AIM from passing through the glomerular filtration barrier and being excreted in urine [34,42]. In AKI, mouse AIM dissociates from IgM and appears in the urine, where it binds to necrotic debris in the tubules and is phagocytosed by tubular epithelial cells expressing KIM-1, thereby promoting recovery from AKI [33]. In contrast, feline AIM does not dissociate from IgM, resulting in persistent cellular debris and impaired recovery from AKI [51]. Thus, we hypothesized that in response to ureteral inflammation, AIM is released into the urine and contributes to tissue repair, but that feline AIM, due to its strong affinity for IgM, behaves pathologically similar to AIM knockout. In this study, three genotypes of mice (fA, koA, and mA groups) were subjected to UUO, and CaOx beads, which serve as substitutes for CaOx stones, were implanted into the dilated ureter to induce ureterolithiasis and develop a feline-like ureteral obstruction model.

At necropsy, both CaOx-implanted (C) and unilateral ureteral obstruction (U) groups showed hydronephrosis in the left kidney. No significant differences were observed in blood biochemical tests among the groups, and no marked abnormalities were detected, suggesting, as reported previously [52], that the right kidney compensated for the function of the left kidney, maintaining normal urination until necropsy. During the study period, there was no notable weight loss, and food and water intake remained adequate, indicating that this model did not substantially reduce the quality of life of the mice, and none of the mice reached the endpoint criteria. However, due to potential complications such as intestinal bleeding, torsion, or adhesions during ureteral manipulation, 4 of 58 mice died before the end of the experiment. While UUO via ligation has been widely used [19,20], this model adds complexity through ureteral incision, bead implantation, and suturing, making it technically more demanding. Although the procedure time must be consistent (12 ± 3 min for ligation and 16 ± 2 min for bead implantation and suturing), we considered the model feasible and broadly applicable with adequate microsurgical training.

Complete or partial obstruction of the ureter, or migration of stones, can cause severe tissue injury to the kidney and ureter [20,53,54]. CaOx stones have a rough surface and cause mucosal damage as they move through the ureter. Tissue injury in the ureter leads to fibrosis, resulting in wall thickening, strictures, or obstruction. Histopathological changes include epithelial desquamation, infiltration of neutrophils, macrophages, lymphocytes, and plasma cells, along with fibroblast proliferation, granulation tissue formation, and obscured boundaries between the muscularis and lamina propria. These changes have been reported previously [21], and similar fibrosis was induced in C57BL/6J mice by ureteral implantation of CaOx beads.

In our study, histopathological examination revealed epithelial desquamation and immune cell infiltration (neutrophils, macrophages, lymphocytes) around CaOx beads in the fA-C, koA-C, and mA-C groups. Although the koA-C group showed a tendency toward higher infiltration scores in the renal parenchyma, pelvis, and ureter, no clear differences in inflammatory cell infiltration were observed among the AIM variants. In site-specific evaluations, the ureter in the CaOx group had the highest infiltration scores compared to the sham group. This suggests that inflammation in the renal parenchyma and pelvis was primarily induced by UUO, while in the ureter, it was due to direct injury from CaOx beads. These inflammatory responses were not significantly influenced by AIM activity.

TGF-β, a pro-fibrotic cytokine produced by macrophages, activates fibroblasts involved in physiological wound repair [55]. However, dysregulated fibroblast activation promotes extracellular matrix (ECM) protein production, rich in collagen from myofibroblasts, leading to fibrosis [55]. In our study, ImageJ-based fibrosis quantification showed that CaOx groups had greater fibrotic areas than sham groups across all genotypes, with the mA group showing the smallest fibrotic area. IHC staining for αSMA, a myofibroblast marker, showed the highest expression in the koA-C group and the lowest in the mA group. These results suggest that AIM suppresses fibrotic progression and that a lack of AIM activation may lead to uncontrolled fibroblast activity and fibrosis. In future studies, we aim to strengthen the proposed mechanism by incorporating the assessment of additional fibrosis markers, such as Col1a1 and TGF-β1.

To confirm AIM expression in ureteral tissue, IHC staining using rabbit polyclonal anti-AIM antibody was performed. Matsuura et al. confirmed that AIM binds to CaOx crystals [42]. AIM contains scavenger receptor cysteine-rich (SRCR) domains: three in mice (SRCR1–3) and three or four in cats (two SRCR1 domains, plus SRCR2 and SRCR3) [51]. SRCR1 is negatively charged, allowing efficient binding to positively charged CaOx crystals [42]. Therefore, we expected that activated AIM would bind to CaOx beads in the ureter. IHC results showed strong AIM positivity around the CaOx crystals in the mA-C group, whereas the fA-C group showed staining similar to the koA-C group. These results demonstrate that in the mouse model, mouse AIM dissociates from IgM and binds to CaOx stones. However, the antibody used in this study is not validated for feline AIM, and its cross-reactivity is currently unconfirmed. Thus, it remains unclear whether feline AIM binds to CaOx crystals under these conditions.

However, IHC staining in this study assessed AIM in tissue rather than its direct binding to intraluminal CaOx beads. The CaOx crystals found in the tissue likely resulted from binding of AIM to intraluminal CaOx beads and subsequent phagocytosis via KIM-1 expressing epithelial cells. Therefore, while we cannot confirm AIM activity in urine, the possibility remains that UUO-induced AKI in the left kidney could have led to AIM dissociation from IgM, filtration through the glomerulus, and excretion into the urine.

The CaOx beads used in this study need further optimization. They are mechanically weak, disintegrate easily, and often deform when in contact with moisture during implantation. In some cases, this led to bead migration into the renal pelvis. However, this could resemble the clinical scenario of renal pelvic stones and may serve as a dual model for ureteral and renal injury. While glyoxylic acid-induced CaOx crystal formation in the kidney has been used in mice [42], it does not represent a fully established stone disease model. In our prior studies, ureteral stone formation sufficient to induce clinical ureterolithiasis was difficult to achieve. Developing more durable CaOx beads may improve the model fidelity for studying ureteral injury caused by stones. Improving bead retention within the ureter would also allow assessment of AIM’s role in intraluminal injury.

In conclusion, this study demonstrates that feline AIM exhibits a distinct phenotype in response to intraluminal ureteral injury compared to mouse AIM. This suggests that AIM plays a role in tissue response to ureterolithiasis, and that feline AIM may contribute to the increased susceptibility to fibrosis observed in cats. Future work using this model will focus on investigating mechanisms and therapies to suppress or prevent fibrosis. This study is the first to demonstrate that feline AIM, characterized by its high affinity for IgM, is associated with increased ureteral fibrosis in response to CaOx-induced injury. These findings provide novel insights into the molecular basis of feline-specific ureteral pathology and fibrosis progression.

## 4. Materials and Methods

### 4.1. Test Animals

18 C57BL/6J mice (CLEA Japan, Tokyo, Japan) (body weight: 22.15 ± 0.93 g, 8 weeks old, male), 18 AIM-knockout C57BL/6J mice (University of Tokyo, Tokyo, Japan) (22.91 ± 1.17 g, 8 weeks old, male) and 18 AIM-felinized C57BL/6J mice (University of Tokyo, Tokyo, Japan) (23.52 ± 1.72 g, 8 weeks old, male) were used (total *n* = 58). During the experimental period, 4 mice died and were excluded from the analysis; thus, 54 mice were included in the final analysis. The animal facility was maintained at a temperature of 22 ± 2 °C, humidity of 40–60%, and a 12 h light/dark cycle. Water and CE-2 feed (CLEA Japan, Tokyo, Japan) were provided ad libitum. The microbiological status of the animals was monitored regularly according to the guidelines of the Japanese Association of Public and Private University Laboratory Animal Facilities. Experiments were approved by the Kitasato University Laboratory Animal Committee (Approval No. 24-019, 1 April 2024) and conducted in accordance with the Guidelines for Laboratory Animals and the Manual for the Care and Management of Laboratory Animals.

#### AIM-Knockout and AIM Felinized C57BL/6J Mice

**Figure 8:**
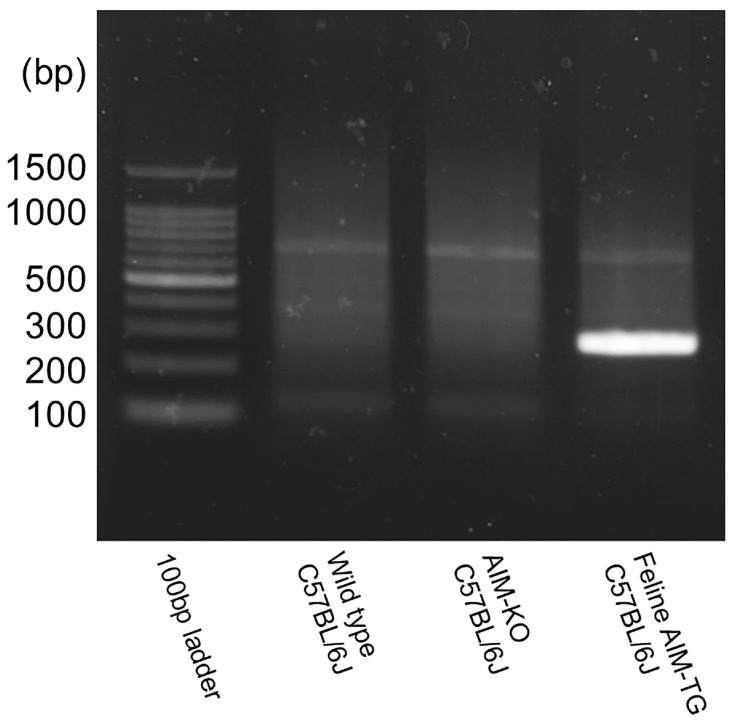
Identification of the feline AIM gene in AIM-felinized mice.

AIM-knockout mice [22] were established by targeted disruption of the murine *Cd5l* gene, which was replaced by a neomycin resistance cassette. The line was subsequently backcrossed onto a C57BL/6J background for more than 15 generations. AIM-felinized mice were generated as previously reported [51]. Briefly, a construct containing feline AIM cDNA under the control of a ~7 kb murine *Cd5l* promoter was prepared within a rabbit β-globin non-coding exon/intron cassette. Transgenic founders were established by pronuclear microinjection of this construct into fertilized C57BL/6 oocytes. To create a “replacement” model, these transgenic founders were subsequently bred with AIM-knockout mice to obtain a line expressing only feline AIM on a murine AIM-deficient background. In this experiment, DNA was extracted from ear fragments at 4 weeks of age, amplified by PCR, and identified as AIM feline C57BL/6 mice by electrophoresis (Figure 8).

fAIM_f: GAGTGGGGCTCTGTCTGC

fAIM_r: TCAAGCATCAGGTAGGGCCAG

### 4.2. Experimental Groups

Each mouse genotype (mA, koA, fA) was divided into two groups: CaOx implantation group (C group): left ureter was implanted with CaOx beads (*n* = 9 per genotype) and Unilateral ureteral obstruction (U group): underwent identical surgery without bead implantation (*n* = 9 per genotype). This yielded six total groups: mA-C, mA-U, koA-C, koA-U, fA-C and fA-U. The experimental group settings are shown in Table 1.

### 4.3. Ureteral Lumen Injury Model

#### 4.3.1. Preparation of a CaOx Beads

**Figure 9:**
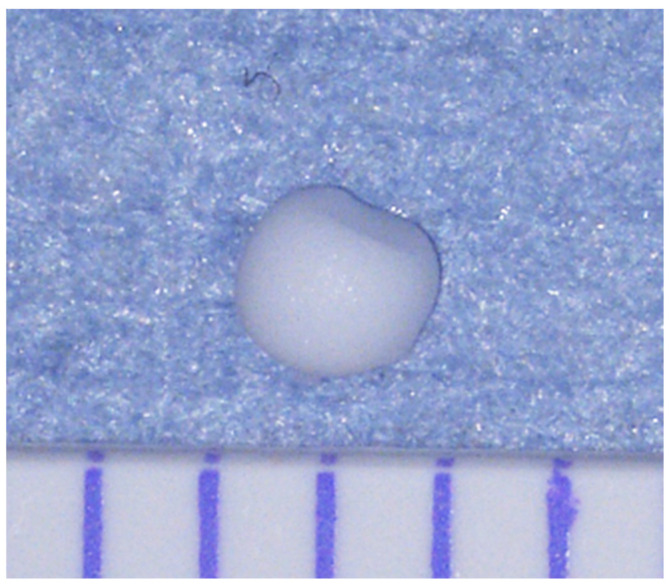
Implanted Calcium oxalate (CaOx) beads. Each division corresponds to 1 mm.

CaOx monohydrate (FUJIFILM Wako Pure Chemicals Corporation, Osaka, Japan) was dissolved in saline solution (Otsuka Pharmaceutical Co., Ltd., Osaka, Japan) to prepare a 1 g/mL solution. A volume of 5 µL of the solution was aspirated using a micropipette and allowed to dry spontaneously at the pipette tip, forming beads. Each bead weighed 4.2 ± 0.12 µg and had a rough surface with face with a diameter of 1.22 ± 0.88 mm (Figure 9).

#### 4.3.2. Anesthesia Protocol, Analgesic Management

Mice were placed in an induction chamber and anesthetized with 3% isoflurane (MSD Animal Health, Tokyo, Japan). After mask application, anesthesia was maintained at 1–2% isofluraine, with respiratory monitoring. Butorphanol tartrate (5 mg/kg) (Vetorufar^®^, Meiji Seika Pharma Co., Ltd., Tokyo, Japan) was administered intraperitoneally as an analgesic.

#### 4.3.3. Ureteral Ligation and Insertion of CaOx Beads

**Figure 10:**
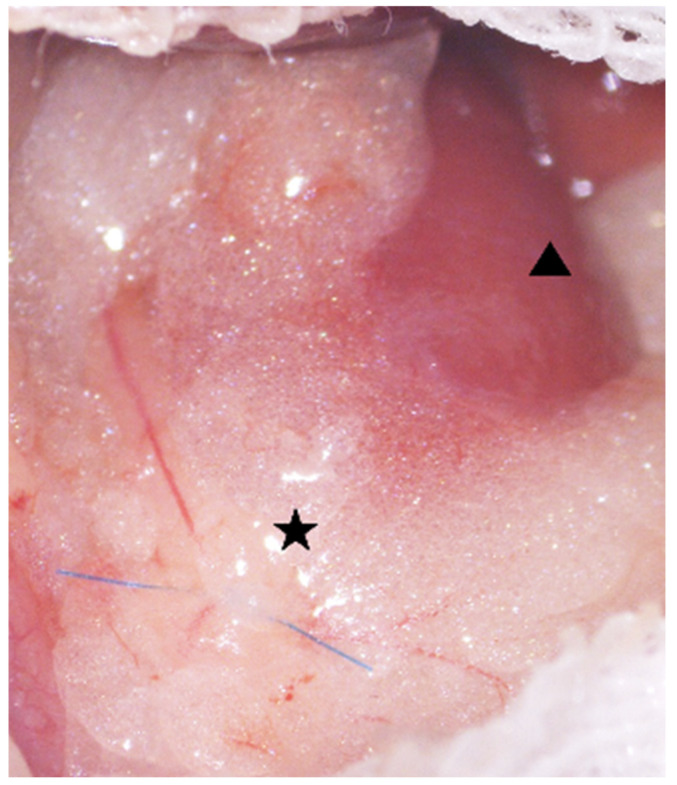
Creation of a UUO model via ligation of the left ureter. ▲: Left Kidney ★: Left Ureter.

The proximal one-third of the left ureter was triple-ligated with 8-0 Prolene^TM^ polypropylene suture (Johnson & Johnson, Inc., Tokyo, Japan) to induce unilateral ureteric obstruction (Figure 10). After 7 days, the dilated ureteral wall was incised, and a CaOx bead (Figure 9) was implanted. The incision was sutured using 10-0 nylon (Keisei Medical Industry Co., Ltd., Tokyo, Japan).

### 4.4. Endpoint Criteria

As humane endpoints, if (1) appetite was attenuated and (2) body weight was reduced by more than 20%, the animals were deeply anesthetized with isoflurane and then euthanized by cervical dislocation.

### 4.5. Blood and Biochemical Tests

On day 14 post-implantation, approximately 0.4 mL of blood was collected from the posterior vena cava and centrifuged (1700× *g*, 15 min, 4 °C). The resulting plasma was measured for blood urea nitrogen (BUN) and creatinine (Cre) concentrations.

### 4.6. Histopathological Examination

#### 4.6.1. Tissue Collection and Sectioning

**Figure 11:**
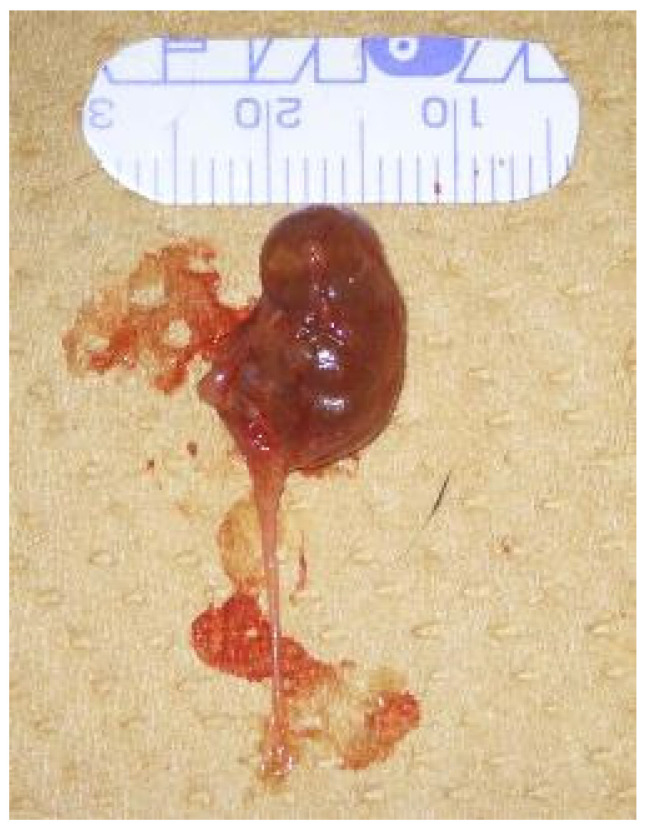
Left kidney and ureter during tissue collection. Each division on the scale marks indicates 1 mm.

Fourteen days after bead implantation, mice were anesthetized using a triple anesthesia protocol: medetomidine hydrochloride (0.75 mg/kg), midazolam (4 mg/kg), and butorphanol tartrate (5 mg/kg). Following anesthesia and exsanguination via the posterior vena cava, both kidneys and ureters were harvested (Figure 11). Tissues were fixed overnight in ALTFiX solution (Pharma Corporation, Tokyo, Japan), paraffin-embedded, and sectioned at 5 µm.

#### 4.6.2. Tissue Staining

Tissue sections were stained with HE and MT stain after normal histological processing. For MT staining, tissue slides were deparaffinized with xylene and alcohol, immersed in an equal volume mixture of 10% potassium dichromate solution/10% trichloroacetic acid solution for 20 min, and stained with iron hematoxylin for 5 min. The cells were then stained with a mixture of equal volumes of 2.5% phosphotungstic acid solution/2.5% phosphomolybdic acid for 40 s, immersed in 0.75% orange G solution for 1 min, and then in a mixture of Ponceau-Kiridin, fuchsin acid, and azofloxin for 25 min. Finally, after staining with 2.5% phosphotungstic acid solution for 7 min, the connective tissues were stained with aniline blue, and the specimens were then stained with alcohol, dehydrated, and sealed.

HE-stained tissue specimens were used to evaluate tissue structure under the microscope. The degree of inflammatory cell infiltration in the kidney, renal pelvis, and ureter was classified as Grade 0–4 (*n* = 6 each; Grade 0: none, Grade 1: slightly infiltrated, Grade 2: somewhat extensively or in several places, Grade 3: extensively infiltrated, Grade 4: almost entirely infiltrated; Figure 2) [56]. The degree of shedding of transitional epithelium and vasodilation was also evaluated.

#### 4.6.3. Fibrosis Quantification

Quantification of fibrosis was performed using MT-stained tissue sections. Each subgroup initially included *n* = 9, but due to tissue damage or poor staining, some samples were excluded from analysis, resulting in a final *n* of 6 for histopathology. For quantification, ImageJ software (version 1.54) (National Institutes of Health, Bethesda, MD, USA) was used to calculate the percentage of fibrotic area per ureteral area. Kidneys and blood vessels were excluded to assess fibrosis in the ureter. For each group (*n* = 6), three random fields per slide per sample were analyzed. The images were binarized, fibrotic areas were segmented, and the percentage of fibrotic area per ureter area was calculated. Analysis was performed by a researcher different from the manuscript authors.

### 4.7. IHC Staining

#### 4.7.1. αSMA (Acta2)

Paraffin-embedded tissue sections (5 µm thick) were heat-treated at 98 °C for 45 min in a 200-fold diluted antigen retrieval solution (ImmunoSaver, Nissin EM Co., Ltd., Tokyo, Japan) to perform antigen retrieval. Next, the sections were incubated in a mixture of 30% hydrogen peroxide (H_2_O_2_) and methanol (100 µL) for 20 min to inactivate endogenous peroxidase. As the primary antibody, 1 µL of rabbit polyclonal anti-αSMA antibody (Funakoshi Co., Ltd., Tokyo, Japan) diluted in 500 µL of phosphate-buffered saline (PBS) was applied, and the slides were incubated at 4 °C overnight. The sections were incubated with the secondary antibody, peroxidase-labeled anti-rabbit IgG polyclonal antibody (Simple Stain Mouse MAX-PO^®^: H2205, Nichirei Bioscience, Tokyo, Japan), at room temperature for 1 h, and then stained with 3, 3-diaminobenzidine (DAB: 040-27001, Fujifilm Wako Pure Chemical Corporation, Tokyo, Japan). The staining time was set to 3 min. The stained sections were imaged using an inverted microscope (ECLIPSE, Nikon, Tokyo, Japan). Three fields of view were imaged per sample, and the number of αSMA-positive cells within each field of view was counted. The ureteral area was measured using ImageJ software, and the number of αSMA-positive cells per µm^2^ were calculated. Due to tissue damage or poor staining, some samples were excluded from analysis. The final *n* values were as follows: Group C: *n* = 4 × 3 fields of view; Group S: *n* = 3 × 3 fields of view.

#### 4.7.2. AIM (CD5L)

Immunohistochemistry for AIM was performed following the same protocol as described in Section 4.7.1. Endogenous peroxidase activity was blocked by incubating tissue in 0.3% H_2_O_2_ in PBS. After rinsing, nonspecific binding was blocked using 10% normal goat serum (Nichirei Bioscience, Tokyo, Japan). The sections were then incubated overnight at 4 °C with a rabbit polyclonal anti-CD5L antibody (GTX37448, Funakoshi Co., Tokyo, Japan; dilution 1:1000). The next day, sections were washed and developed with DAB for 90 s to visualize AIM expression. For analysis, images were captured using a Nikon ECLIPSE inverted microscope (Nikon, Tokyo, Japan). The AIM-positive area surrounding the CaOx crystals was quantified using ImageJ software (NIH, Bethesda, MD, USA) and expressed as a percentage of the total crystal area. Due to tissue damage or poor staining, some samples were excluded from analysis, resulting in a final *n* of 3 per group.

### 4.8. Statistical Analysis

All statistical analyses were performed using JMP Pro 17 (SAS Institute, Cary, NC, USA). Normality was assessed using the Shapiro–Wilk test, and homogeneity of variances was evaluated using Levene’s test. Depending on the results, data were analyzed by one-way ANOVA or Kruskal–Wallis test. Post hoc comparisons were performed using Tukey–Kramer or Steel–Dwass tests when significant differences were detected. A *p*-value < 0.05 was considered statistically significant. Error bars indicate standard deviation.

## Figures and Tables

**Table 1:** Experimental Groups.

		Calcium Oxalate Group	Unilateral Ureteral Obstruction
		C Group	U Group
C57BL/6	mA	mA-C Group	mA-U Group
Mouse AIM-KO-C57BL/6	koA	koA-C Group	koA-U Group
AIM Felinized C57BL/6	fA	fA-C Group	fA-U Group

## Data Availability

All data gene rated or analyzed during this study are included in this published article.

## Supplementary Materials

The following supporting information can be downloaded at: https://www.mdpi.com/article/10.3390/ijms26189117/s1.

## Abbreviations

The following abbreviations are used in this manuscript:

AIM	Apoptosis inhibitor of macrophage
CKD	Chronic kidney disease
AKI	Acute kidney injury
CaOx	Calcium oxalate
UUO	Unilateral ureteral obstruction
HE	Hematoxylin and Eosin
MT	Masson’s Trichrome
αSMA	Alpha-smooth muscle actin
BUN	Blood urea nitrogen
CRE	Creatinine
TGF-β	Transforming growth factor-beta
ECM	Extracellular matrix
SRCR	Scavenger receptor cysteine-rich
