# Supplementary material for: Apoptosis Inhibitor of Macrophage (AIM) Modulates Calcium Oxalate-Induced Ureteral Fibrosis in AIM-Felinized Mice
Source: Int J Mol Sci. 2025 Sep 18;26(18):9117. doi: 10.3390/ijms26189117 (PMC12471175; doi:10.3390/ijms26189117)
Supplement: Supplementary file 1 [file ijms-26-09117-s001.zip › ijms-3836487-supplementary.pdf]

Figure Legends for Supplementary Figures

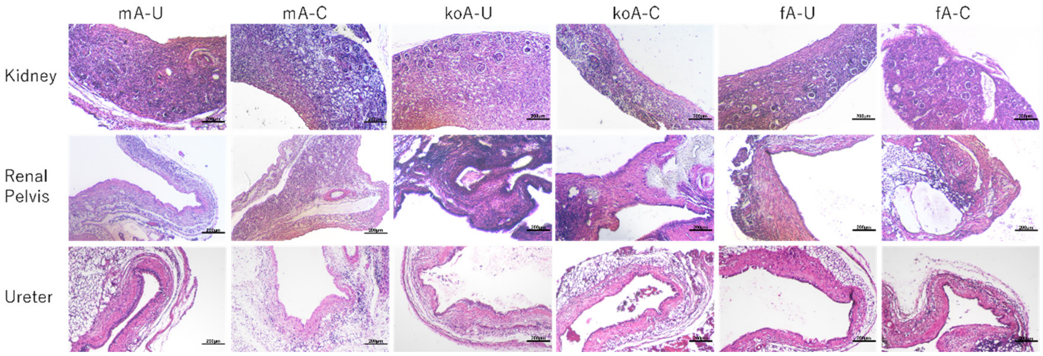

Supplementary Figure S1. Representative images of each group used for evaluating inflammation by hematoxylin and eosin (HE) staining. The scale bar represents 200μm.

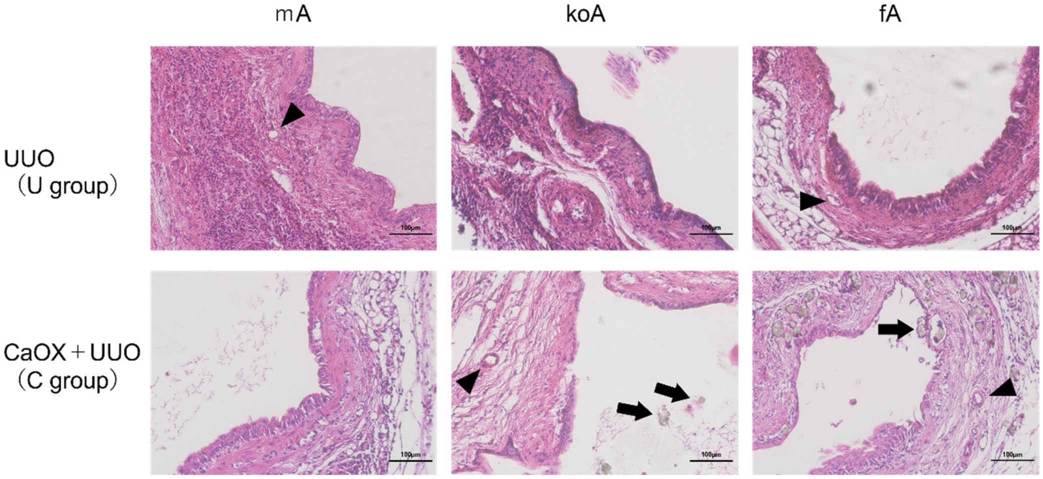

Supplementary Figure S2. Representative HE-stained images (×100) of ureteral tissue.

Slight inflammatory cell infiltration and vascular dilation (arrowheads), and exfoliation of the urothelium caused by CaOx beads (arrows) are observed.
